# Supplementary figures and images for: Physiological Changes of Arabica Coffee under Different Intensities and Durations of Water Stress in the Brazilian Cerrado
Source: Plants (Basel). 2022 Aug 25;11(17):2198. doi: 10.3390/plants11172198 (PMC9460576; doi:10.3390/plants11172198)

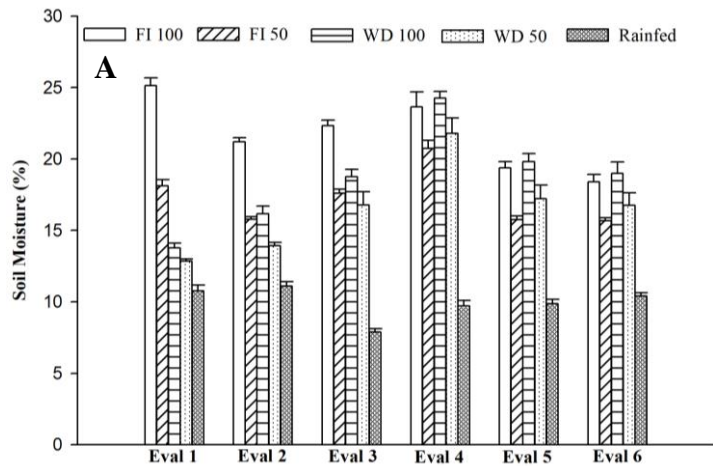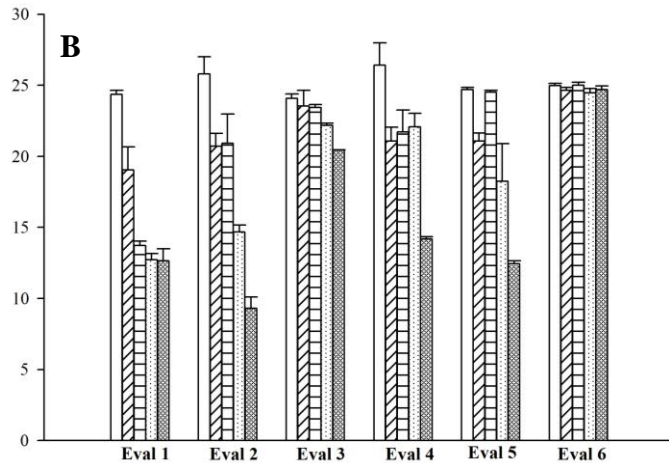

Supplement: Supplementary file 1 [file plants-11-02198-s001.zip › Figure S2.pdf]

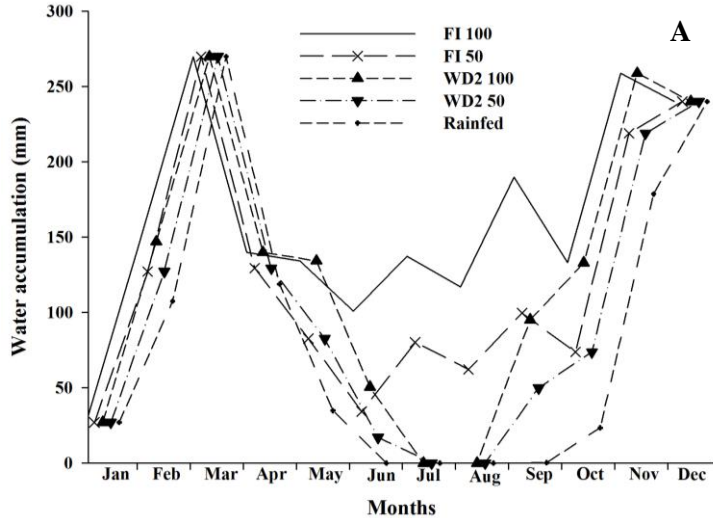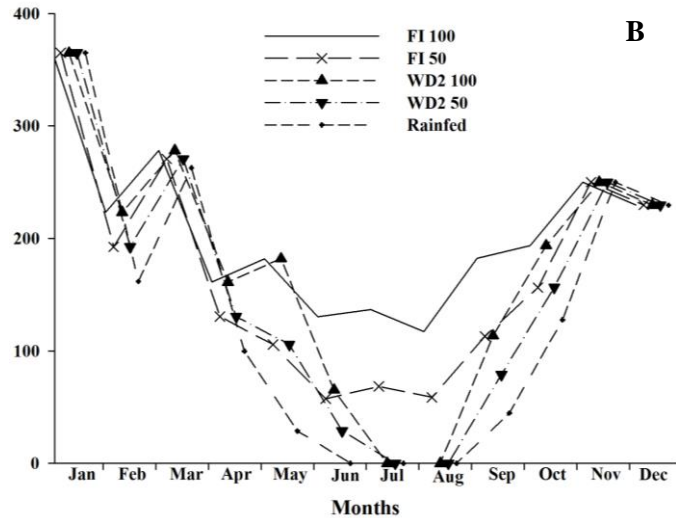

Supplement: Supplementary file 1 [file plants-11-02198-s001.zip › Figure S4.pdf]
