# Supplementary material for: Physiological Changes of Arabica Coffee under Different Intensities and Durations of Water Stress in the Brazilian Cerrado
Source: Plants (Basel). 2022 Aug 25;11(17):2198. doi: 10.3390/plants11172198 (PMC9460576; doi:10.3390/plants11172198)
Supplement: Supplementary file 1 [file Figure S1.pdf › Figure S1.pdf]

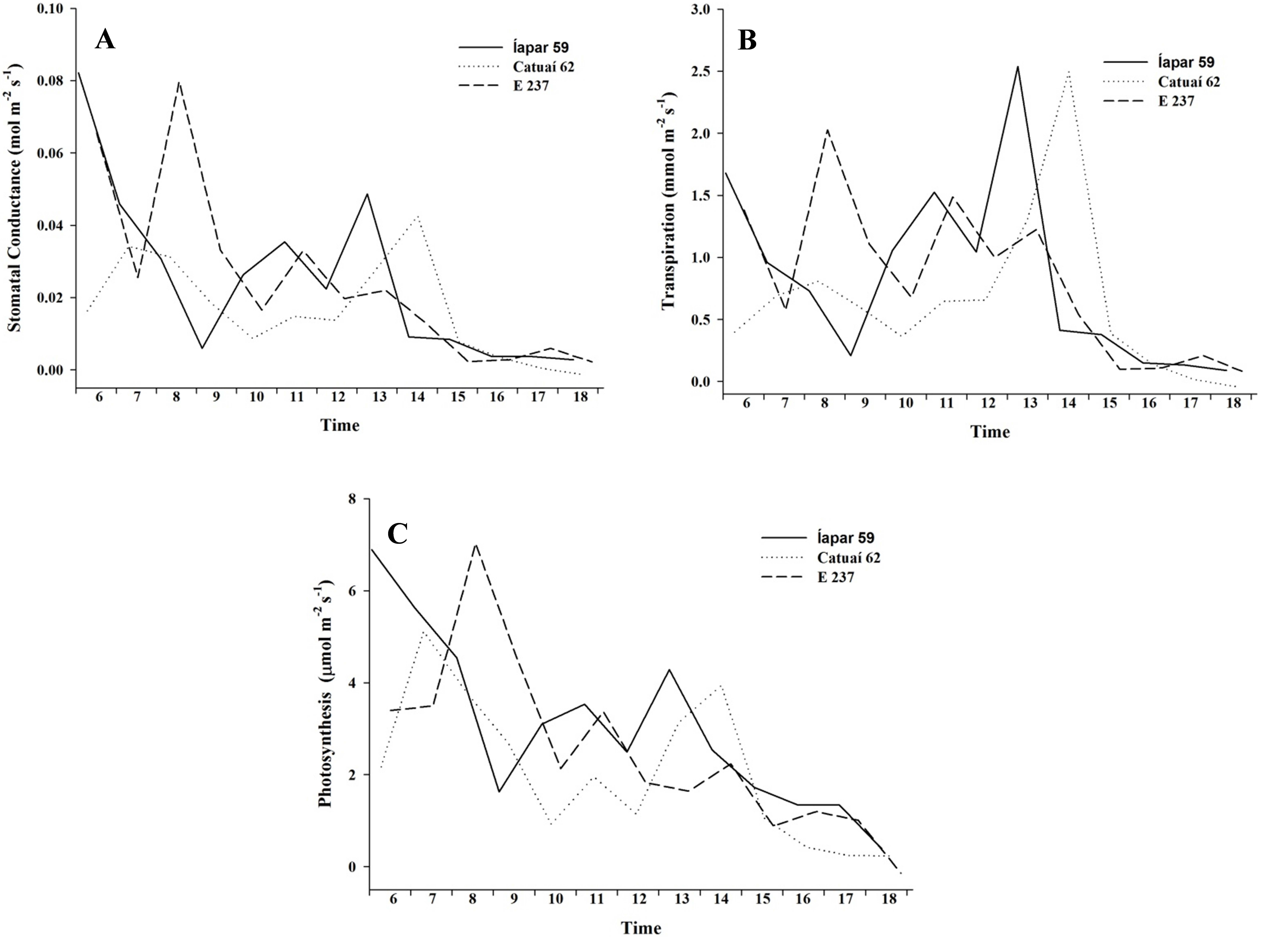
A
Stomatal Conductance (mol m-2 s-1)
0.10
0.08
0.06
0.04
0.02
0.00
6 7 8 9 10 11 12 13 14 15 16 17 18
Time
Íapar 59
Catuaí 62
E 237
B
Transpiration (mmol m-2 s-1)
3.0
2.5
2.0
1.5
1.0
0.5
0.0
6 7 8 9 10 11 12 13 14 15 16 17 18
Time
Íapar 59
Catuaí 62
E 237
C
Photosynthesis (µmol m-2 s-1)
8
6
4
2
0
6 7 8 9 10 11 12 13 14 15 16 17 18
Time
Íapar 59
Catuaí 62
E 237
