# Supplementary material for: Physiological Changes of Arabica Coffee under Different Intensities and Durations of Water Stress in the Brazilian Cerrado
Source: Plants (Basel). 2022 Aug 25;11(17):2198. doi: 10.3390/plants11172198 (PMC9460576; doi:10.3390/plants11172198)
Supplement: Supplementary file 1 [file Figure S3.pdf › Figure S3.pdf]

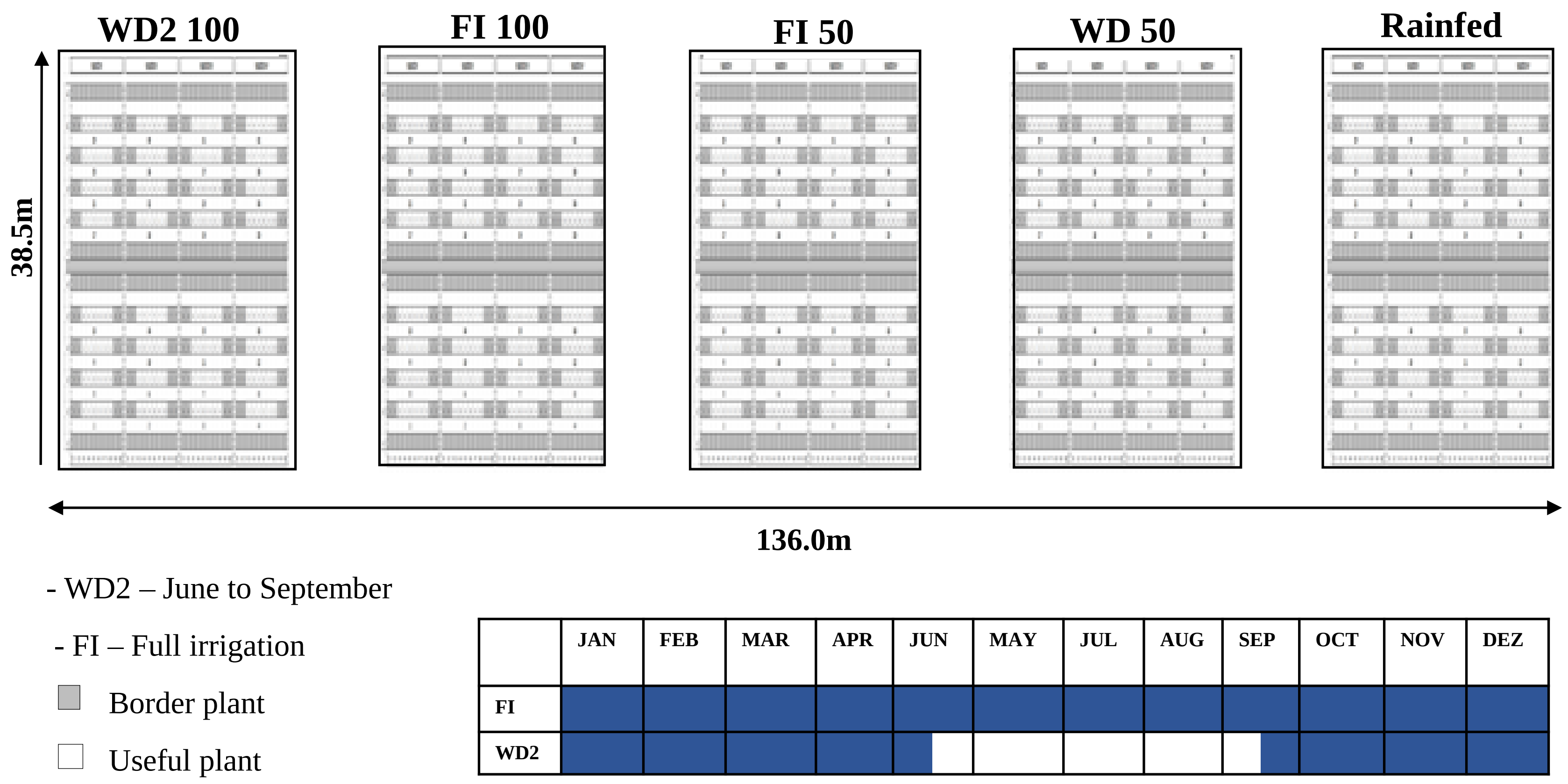
WD2 100
FI 100
FI 50
WD 50
Rainfed
38.5m
136.0m
- WD2 – June to September
- FI – Full irrigation
Border plant
Useful plant
JAN
FEB
MAR
APR
JUN
MAY
JUL
AUG
SEP
OCT
NOV
DEZ
FI
WD2
